# Preparation of Advanced Carbon Anode Materials from Mesocarbon Microbeads for Use in High C-Rate Lithium Ion Batteries

**DOI:** 10.3390/ma8063550

**Published:** 2015-06-17

**Authors:** Ming-Dar Fang, Tsung-Han Ho, Jui-Pin Yen, Yu-Run Lin, Jin-Long Hong, She-Huang Wu, Jiin-Jiang Jow

**Affiliations:** 1 Department of Chemical and Materials Engineering, National Kaohsiung University of Applied Sciences, Kaohsiung 80778, Taiwan; E-Mails: fang@e-cscc.com.tw (M.-D.F.); thho@cc.kuas.edu.tw (T.-H.H.); 2 China Steel Chemical Corporation, Kaohsiung 80245, Taiwan; E-Mails: yen@e-cscc.com.tw (J.-P.Y.); 02454@e-cscc.com.tw (Y.-R.L.); 3 Department of Materials and Optoelectronic Science, National Sun Yat-Sen University, Kaohsiung 80424, Taiwan; E-Mail: jlhong@mail.nsysu.edu.tw; 4 Department of Materials Engineering, Tatung University, Taipei 80104, Taiwan; E-Mail: shwu@ttu.edu.tw

**Keywords:** mesocarbon microbeads, soft carbon, anode materials, high C-rate, lithium ion battery, charge rate capability

## Abstract

Mesophase soft carbon (MSC) and mesophase graphite (SMG), for use in comparative studies of high C-rate Lithium Ion Battery (LIB) anodes, were made by heating mesocarbon microbeads (MCMB) at 1300 °C and 3000 °C; respectively. The crystalline structures and morphologies of the MSC, SMG, and commercial hard carbon (HC) were investigated by X-ray diffraction, transmission electron microscopy, scanning electron microscopy, and Raman spectroscopy. Additionally, their electrochemical properties, when used as anode materials in LIBs, were also investigated. The results show that MSC has a superior charging rate capability compared to SMG and HC. This is attributed to MSC having a more extensive interlayer spacing than SMG, and a greater number of favorably-oriented pathways when compared to HC.

## 1. Introduction

Light weight lithium ion batteries (LIBs) have received increasing interest in recent years due to their high energy densities, long cycle lives, and environmental friendliness. They have become important power sources in consumer electronic products, such as cellular phones and laptop computers. Currently carbon materials are generally used as anode materials for LIBs due to their small surface change, structural stability during cycling, high energy density, and abundance [1]. Carbon materials can be classified in several ways. They can be divided into graphitizable carbons and non-graphitizable carbons, according to differences in the graphitization process, or they can be distinguished as soft carbons, hard carbons, and graphite with different degrees of graphitization [2,3]. Among these carbons, artificial graphite and modified natural graphite are the most commonly chosen carbon anode materials for commercial applications due to their high reversibility. Despite their commercial success, the use of LIBs as a source of electricity for power tools and in the automotive industries is still quite limited, due at least in part to stress generation that results in fracturing/disintegration of the electrode during Lithium ion (Li**^+^**) intercalation and de-intercalation.

The crystalline layered structure of graphitic carbon enables the insertion of Li^+^ to achieve high capacities. However, fully exploiting the potential of crystalline graphite in LIBs remains problematic, due to the capacity decrease that accompanies repeated high C-rate charge-discharging. It has been suggested that the slow solid-state diffusion of Li^+^ within the graphitic anode materials limits the high C-rate capability of both LIBs [4], and amorphous carbon materials (e.g., coke), by having a small crystalline size and a wide interlayer distance, that results in a higher charge-discharge rate, due to the fast diffusion of Li^+^ within their disordered structures [5,6]. However, amorphous carbon materials also show poor capacity retention during their cycle life due to pulverization of the particles, variations in surface morphology, large voids, disordered non-crystallized sites, random lattice structures and contamination from metallic impurities that causes compositional changes during cycling. To reduce the disadvantages of these two types of carbon materials, *i.e.*, graphitic and amorphous [7,8], appropriate combinations, comprising both crystalline and disordered materials, were investigated as potential anode materials for high power applications [9].

In this work, mesophase soft carbon (MSC); mesophase graphite (SMG), prepared from mesocarbon microbeads (MCMBs); and a commercial hard carbon (HC) were selected as high C-rate LIBs anode materials. The three anode materials were assembled in coin-type cells for capacity retention studies using various C-rates for charging and discharging. The results obtained were correlated with the physical properties of the carbonaceous materials obtained from XRD, scanning and transmission electron microscopic (SEM and TEM), and Raman studies. 

## 2. Experimental 

### 2.1. Materials and Instrumentations 

A commercial coal tar pitch was subjected to thermal and chemical treatments to yield MCMBs, which were then either carbonized at 1300 °C for 1 h under an argon atmosphere with a heating rate of 10 °C·min^−1^ to yield low crystallinity MSC, or following the initial treatment, they received additional heat treatment at 3000 °C thereby producing SMG. HC (Kureha Battery Materials Japan Co., Tokyo, Japan) was used as an alternative anode material for comparison purposes. Table 1 lists the properties, of SMG, HC and MSC, including tap and true densities, particle sizes, specific surface areas (SSA). 

**Table 1:** Chara+9cteristics of the mesophase graphite (SMG), hard carbon (HC), and mesophase graphite (MSC) anode materials.

Sample	True density ^a^ (g·cm^−3^)	Tap density ^b^ (g·cm^−3^)	Particle size ^c^ (D_50_, μm)	SSA ^d^ (m^2^·g^−1^)
SMG	2.23	1.22	8.31	2.63
HC	2.06	0.92	10.53	3.40
MSC	2.08	1.10	8.50	2.10

^a^ measured by Micromeritics AccuPyc 1330 Pycnometer; ^b^ measured by Quanta Chrome Auto Tap; ^c^ examined by Malvern Mastersizer S; ^d^ Specific surface area determined by Micromeritics TriStar 3000.

The morphologies of the prepared MSC and SMG were observed with a SEM (Jeol JSM-6510, JEOL Ltd., Tokyo, Japan) and a TEM (Jeol-2010, JEOL Ltd., Tokyo, Japan). The lattice parameters of these anode materials were determined from conventional powder X-ray diffraction patterns, collected with an X-ray diffractometer (Rigaku MiniFlexII, Rigaku Corp., Tokyo, Japan) with CuKα radiation (2θ between 10° and 80°, scan rate 5° min^−1^). The average crystallite dimensions, La in the *a*-axis, and Lc in the *c*-axis direction of the graphite plane, were estimated from the half-line widths of the (110) and (002) diffraction peaks, respectively. Raman spectra of the candidate anode materials (in powder form) were recorded with a Horiba Ihr 550 model in the range 1000–3000 cm^−1^. The average ID/IG ratio evaluated from the relative integrated intensities of the D-band (defect-induced band near 1345 cm^−1^) and the G-band (graphite-related band at 1550–1605 cm^−1^), has been used to quantify the anode materials’ crystallinity. 

### 2.2. Electrochemical Analysis

Anode materials (94 wt %) were mixed with PVDF (5 wt %, Solef type 6020), Super S (1%, Timcal) and NMP (N-methyl-pyrrolidone, ISP) to form slurries, and then smeared on Cu foil using a doctor blade coater, vacuum dried overnight at 120 °C and roll pressed to obtain electrodes. The electrodes were punched into 13.0 mm (ID) disk electrodes and assembled into CR2032 coin-type cells with a Li counter electrode, LiPF_6_ (1 M) dissolved in EC/DEC (1/1 v/v, Mitsubishi Chemical) electrolyte, and separator (Celgard 2325, Celgard Ltd., Charlotte, NC, USA) in an argon-filled glove box. The cycling performance of the coin cells was examined using a Maccor 4000 multichannel battery tester at 25 °C. The cells were charged at a constant current (CC) density of 0.4 mA·cm^−2^ (0.2 C rate) to 0.001 V, which was followed by charging in constant voltage (CV) mode with cutoff current of 0.02 C and then discharged at 0.2 C to 1.5 V for two cycles, prior to the cycling test. The cells were then cycled between cutoff voltages of 0.001 and 1.5 V by charging in constant current-constant voltage (CC-CV) mode at 0.2 C, as described above, and then discharging sequentially (1 charge/discharge per cycle) at various C-rates (0.2, 0.5, 1, 2, and 5 C). The cells were cycled between cutoff voltages of 0.001 and 1.5 V by charging in CC-CV mode at various C-rates (*i.e.*, 0.2, 0.5, 1, 2, and 5 C) to 0.001 V with a 0.02 C cutoff current followed by consecutively discharging (CC at 0.2 C to 1.5 V).

## 3. Results and Discussion

**Figure 1:**
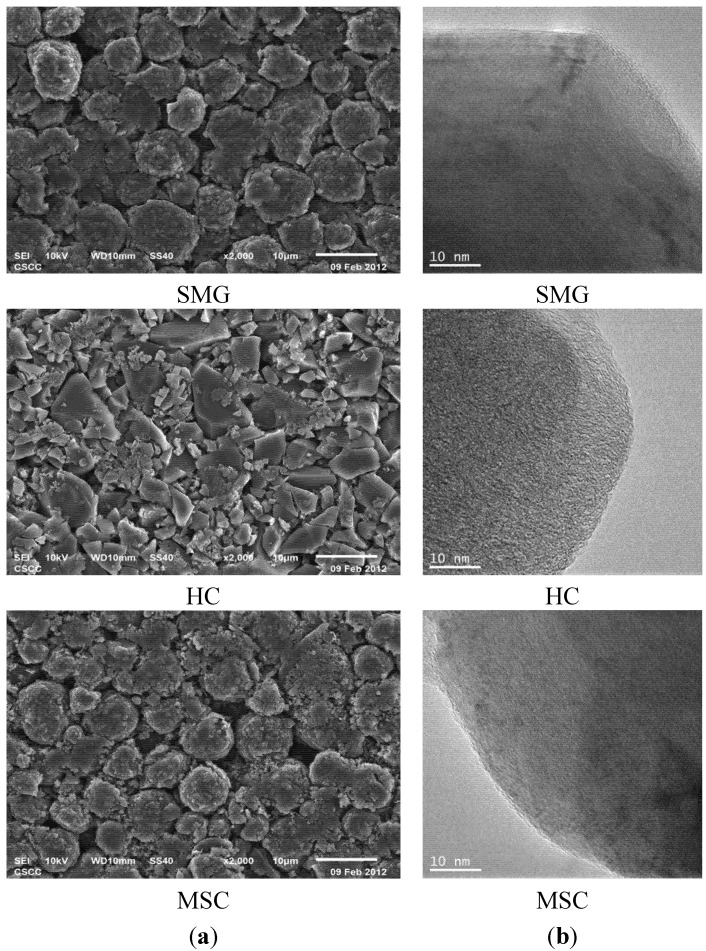
(**a**) SEM and (**b**) TEM images of SMG, HC, and MSC.

SEM and TEM images of SMG, HC and MSC are shown in Figure 1. 

From the SEM images, it is apparent that MSC and SMG are spherically shaped with average diameters of 8 to 9 μm; however, there are more fine fragments on the surfaces of the carbonized MSC materials than on the high-temperature graphitized SMG. In contrast to the spherical shapes observed for MSC and SMG, the HC sample shows polyhedral flake plates with a lot of small fragments with dimensions of 1–2 μm. This suggests the reasons for the higher SSA (3.4 m^2^·g^−1^), compared to those for the spherical SMC and MSG samples (*i.e.*, 2.63 and 2.10 m^2^·g^−1^, respectively), see Table 1. From the TEM images, obvious and long-range layers are observed with SMG, whereas less distinct, short, layered structures are found on the MSC particles’ surfaces. This suggests that the SMG sample has a higher crystallinity and higher-ordered layered structures with unified orientation, compared to MSC. The TEM image of hard carbon reveals that the sample consists of short-layered grains with staggered stacking. The high true and tap densities for SMG (Table 1) can be attributed to the highly crystalline structure that results from high-temperature heating process. 

**Figure 2:**
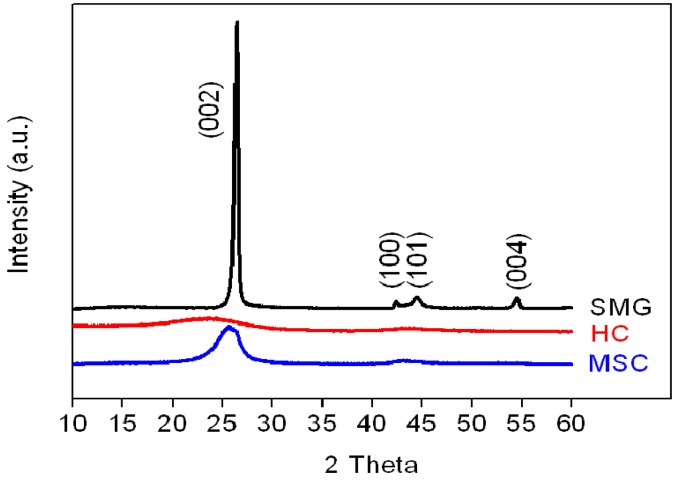
XRD patterns of SMG, HC, and MSC anode materials.

The X-ray diffraction patterns of the three anode materials are shown in Figure 2.

Compared to the weak and broad (002) peaks of the HC and MSC samples at 2θ = 26.8°, the corresponding diffraction peak of SMG has a relatively high intensity, indicating that high-temperature heat treatment at 3000 °C markedly increases the crystallinity of the SMG. The calculated interlayer spacings (d002 value) of SMG (0.3368 nm) are smaller than those of MSC and HC (0.3478 and 0.3794 nm, respectively, as listed in Table 2). For the HC and MSC materials, the weak and broad (002) and (100) diffraction peaks suggest the presence of small domains of coherent and parallel stacked graphene sheets.

**Table 2:** Structural parameters of the SMG, HC and MSC anode materials estimated from XRD patterns and Raman spectroscopy.

Sample	d(002) (nm) ^a^	Lc (nm) ^b^	La (nm) ^c^	*R* value ^d^
SMG	0.3368	20.7	45.1	0.20
HC	0.3794	1.0	4.0	1.09
MSC	0.3478	3.2	5.1	0.90

XRD parameters: ^a^ lattice distance d(002); ^b^ thickness of edge plane Lc; ^c^ length of basal plane La, performed with Rigaku MiniFlexII; ^d^ *R* value (ID/IG), determined form the Raman spectra.

**Figure 3:**
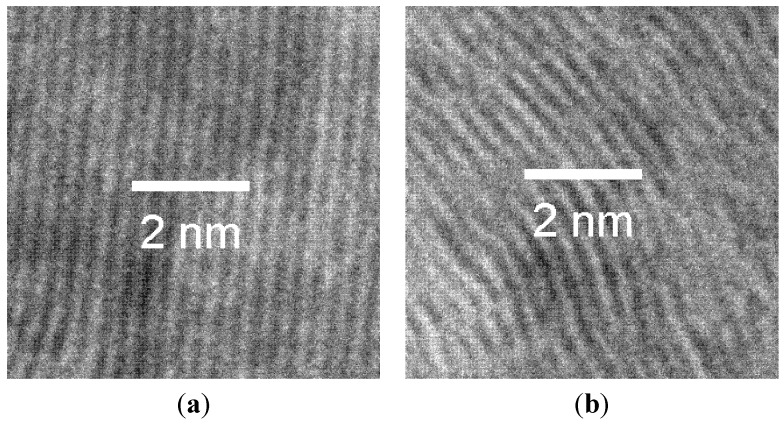
TEM images of (**a**) SMG and (**b**) MSC.

The values of the Lc parameters, evaluated from the line widths of the (002) peak (used to determine the relative degree of crystallinity of SMG, HC and MSC), are 20.7 nm, 1.0 nm and 3.2 nm, respectively. The large Lc value for SMG can be attributed to its highly stacked parallel crystallinity, which is consistent with its ordered structure; while the graphene layers of MSC are observed in broad and curved structural units. These results are consistent with the TEM images for SMG and MSC, see Figure 3a,b.

**Figure 4:**
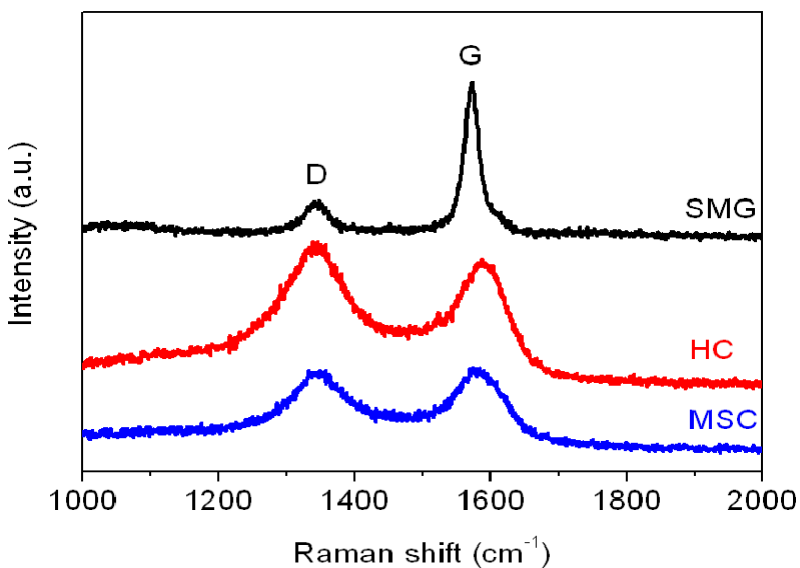
Raman spectra plot of SMG, HC, and MSC anode materials.

The in-plane characteristics of the anode materials were characterized from the Raman spectra, as shown in Figure 4. 

The average ID/IG ratio (*R* value in Table 2) evaluated from the integrated intensities of the D-band (defect-induced mode, near 1350 cm^−1^) and G-band (graphite-related mode, at 1550–1605 cm^−1^) were used to determine the degree of crystallinity of the anode samples. The high-temperature graphitized SMG shows a relatively lower *R* (0.20) compared to HC and MSC (1.09 and 0.9, respectively). The low *R* value is a characteristic of a well-developed (high crystallinity) graphite structure. 

**Figure 5:**
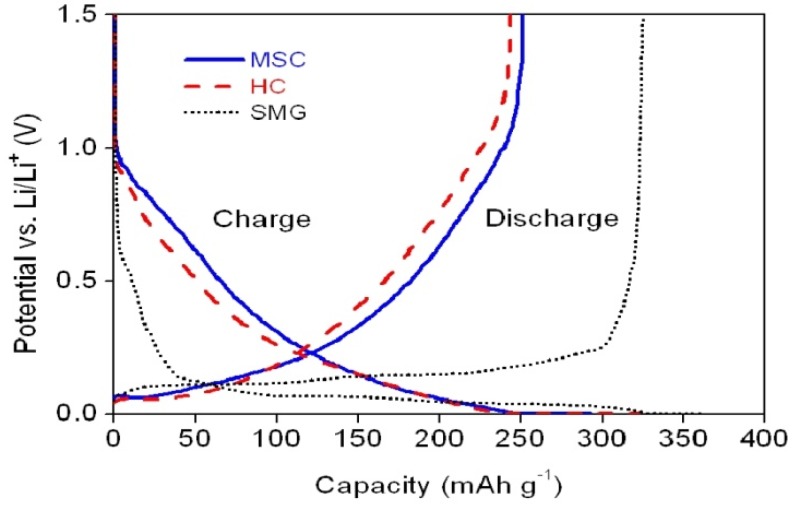
Initial charge/discharge curves of the coin-type cells with various anode materials.

The initial charge and discharge profiles of the coin-type cells prepared with SMG, MSC and HC as anode materials are shown in Figure 5.

During the course of charging, the intercalation of Li^+^ ion in the cell with SMG anode starts at approximately 0.5–0.8 V *vs*. Li/Li^+^, due to the irreversible formation of a solid electrolyte interface layer on the carbon surface resulting from electrolyte decomposition and subsequent reduction with Li^+^ ions [10,11]. There follows a long plateau, due to Li^+^ ions intercalating at voltages lower than 0.2 V *vs.* Li/Li^+^. The cells with MSC and HC anodes show sloping potential profiles starting as 1 V *vs*. Li/Li^+^. This behavior is attributed to Li^+^ ion intercalation into the highly distorted structure and variously distributed active sites [12,13]. The SMG cell exhibits both a higher initial discharge capacity (325.1 mAh·g^−1^) and a greater coulombic efficiency (90%) than either the MSC and HC cells. Although the MSC and HC cells show very similar charge/discharge characteristics, the MSC anode has both a slightly higher initial capacity (248.5 mAh g^−1^) and a greater coulombic efficiency (85.2%) than the HC anode (243.5 mAh g^−1^ and 75.4%, respectively), as shown in Table 3. 

**Table 3:** Initial charge/discharge capacities and coulombic efficiencies of the cells with various anode materials.

Anode materials	Initial charge capacity ^a^ (mAh g^−1^)	Initial discharge capacity ^a^ (mAh g^−1^)	Initial coulombic efficiency ^b^ (%)
MSC	291.6	248.5	85.2
HC	322.9	243.5	75.4
SMG	361.2	325.1	90.0

^a^ measured at 0.2 C; ^b^ calculated by: Initial discharge capacity/Initial charge capacity ×100%.

The discharge curves of carbonaceous anodes have been separated into 3 regions [14,15,16]; the discharge plateau between 0 V and 0.12 V attributed to Li^+^ de-intercalation from micropores among the stacked graphene layers; the sloping curve between 0.12 V and 0.8 V, due to the de-intercalation of Li^+^ intercalated between turbostratically disordered carbonaceous interlayers; and the sloped curve at a potential higher than 0.8 V, which corresponds to the de-intercalation of Li^+^ covalently bound to the edge sites of the graphene layer or large numbers of disordered micropores [17,18]. HC has the lowest initial coulombic efficiency among the samples, which may be due to the irreversible incorporation of Li^+^ in non-crystallized sites, or defect structural voids [19], while MSC shows a lower charge and a higher discharge capacity than HC in the initial cycle.

**Figure 6:**
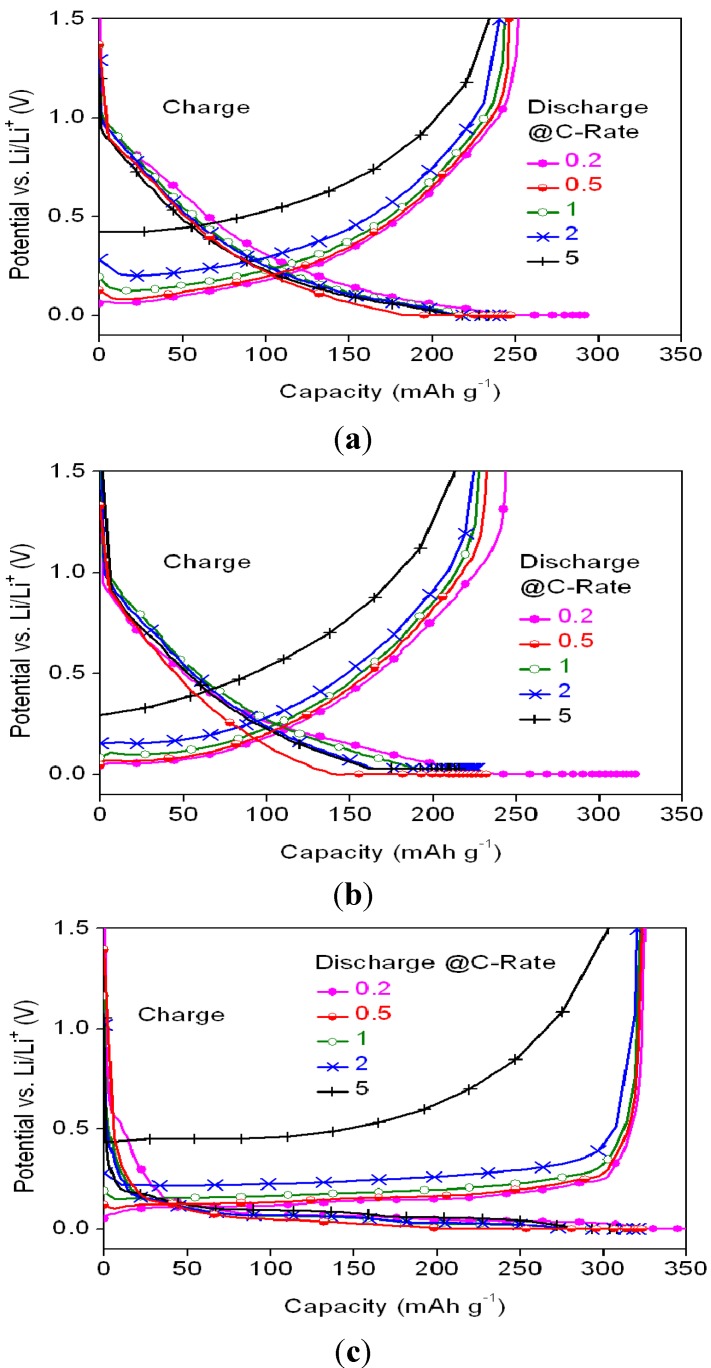
Potential profiles of the cells with (**a**) MSC, (**b**) HC, and (**c**) SMG anode materials at 0.2 C charge rate and various discharge C-rates.

The charge/discharge profiles of different samples are shown in Figure 6, while the ratios of the discharge capacities at various C-rates (relative to discharge at 0.2 C) for the various anode materials are listed in Table 4.

**Table 4:** Discharge capability ratios of the MSC, HC and SMG anode materials at various C-rates.

Samples	Discharge capability ratio relative to 0.2 C (%)			
	0.5 C	1 C	2 C	5 C
MSC	98.0	96.8	95.1	93.2
HC	95.1	93.5	92.1	87.4
SMG	99.4	99.1	98.5	93.1

**Figure 7:**
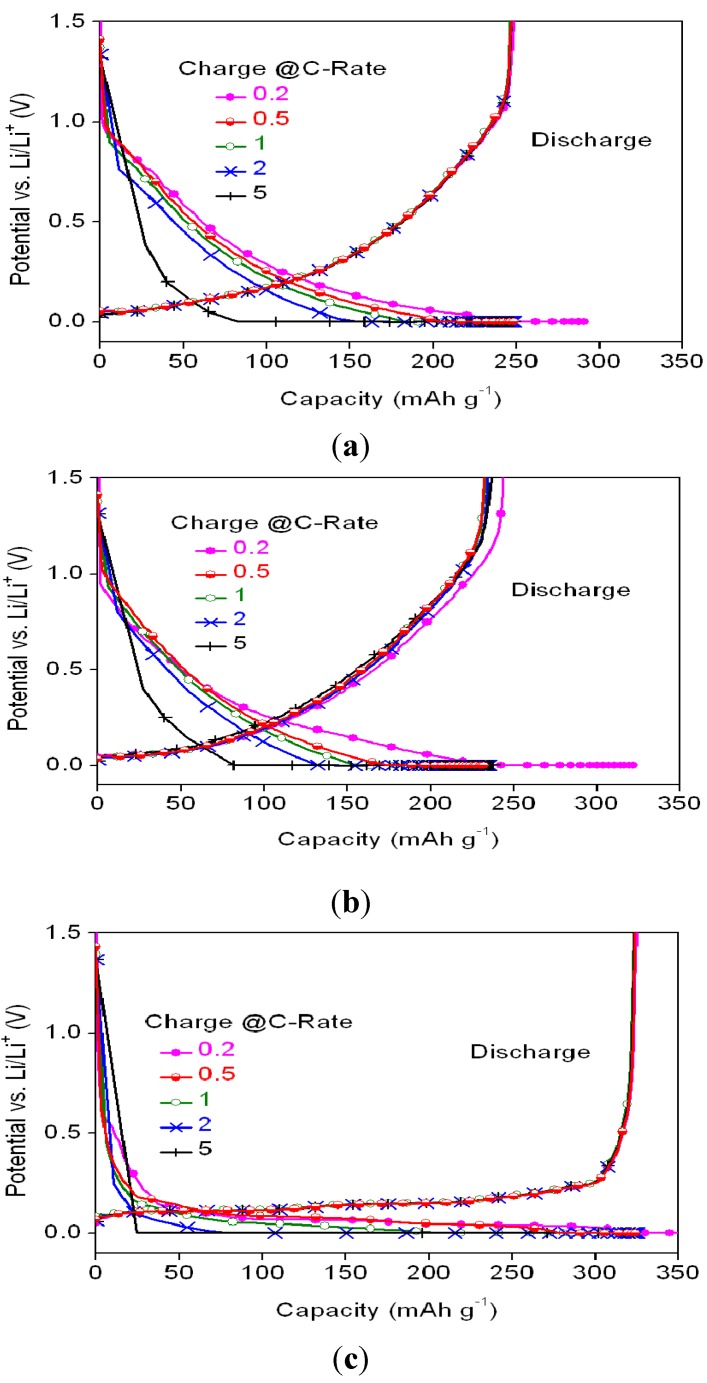
Potential profiles of the cell with (**a**) MSC, (**b**) HC, and (**c**) SMG anode materials at various charge C-rates and 0.2 C discharge rate.

SMG and MSC show higher discharge rate capabilities than HC. The irreversible capacity loss of HC at voltages lower than 0.04 V *vs*. Li^+^/Li was attributed to lithium plating on the carbon surface and into the macropores [20]. Of the three samples, SMG shows the highest reversible capacities at various discharge C-rates, with most of its capacity being seen with voltages lower than 0.5 V.

**Figure 8:**
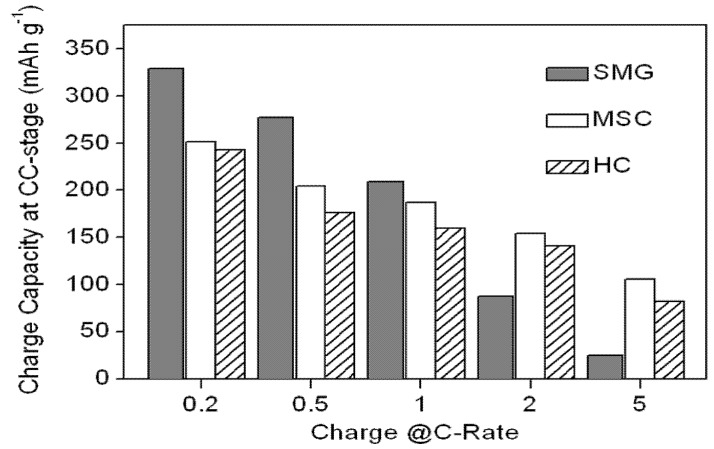
Plots of CC-stage charging capacity of the MSC, HC and SMG anode materials at various C-rates.

In addition to the discharge capability ratios of the MSC, HC and SMG at various C-rates, the charge capacity ratios of the anode materials at various C-rates were also determined. The charge/discharge profiles are shown in Figure 7, while the CC-stage charging capacity and the capacity ratios at various C-rates are listed in Figure 8 and Table 5, respectively.

**Table 5:** CC-stage charging capability ratio for the MSC, HC and SMG anode materials at various C-rates.

Sample	CC-stage charge capacity at 0.2 C (mAh g^−1^)	Charging capability ratio relative to 0.2 C (%)			
		0.5 C	1 C	2 C	5 C
MSC	251.5	81.2	74.4	61.2	42.0
HC	242.4	72.8	65.7	58.2	33.8
SMG	329.0	84.3	63.5	26.7	7.4

Of the three anode materials, SMG shows the greatest charging capacity decrease at the CC-stage. Most of its charging capacity loss occurs when the charging C-rate is higher than 2 C. Though the loss can be compensated for during the CV-stage, the charge capacity in the CV region cannot be fully utilized in the real cell because the potential of the CV-stage is close to the reduction potential of Li^+^, thereby promoting lithium-dendrite deposition during charging [21].

## 4. Conclusions 

The characteristics of various carbon/graphite anode materials, *i.e.*, MSC, SMG and HC, together with the performance of LIBs incorporating these materials were systematically examined. The results show that the soft carbon MSC (carbonized at 1300 °C) has the most promising charging and discharging capability when compared to SMG (graphitized at 3000 °C) and HC. This is attributed to it having a more extensive crystalline carbon interlayer spacing with better oriented paths that favor diffusion of Li^+^ ions within the electrodes, thereby leading to a better discharge rate capability. 

The graphitized materials SMG shows a high initial coulombic efficiency and a reversible capacity at a low charging C-rate, but an inferior charging performance at high C-rates, due to its highly-crystalline layered structure with narrow interlayer spacing d(002) and the long basal plane within the crystalline graphite domains. HC has a better charging rate capability at the CC-stage, due to its large interlayer spacing; however, local disorder and the spacious turbostratic graphite structures lead to a large irreversible capacity in the initial cycle. 

Taken together, the facts suggest that the soft carbon materials produced at low temperature represent the most economic option, of the three anode materials, for high C-rate LIBs.
